# Individualized estimation of human core body temperature using noninvasive measurements

**DOI:** 10.1152/japplphysiol.00837.2017

**Published:** 2018-02-08

**Authors:** Srinivas Laxminarayan, Vineet Rakesh, Tatsuya Oyama, Josh B. Kazman, Ran Yanovich, Itay Ketko, Yoram Epstein, Shawnda Morrison, Jaques Reifman

**Affiliations:** ^1^Department of Defense Biotechnology High Performance Computing Software Applications Institute, Telemedicine and Advanced Technology Research Center, United States Army Medical Research and Materiel Command, Fort Detrick, Maryland; ^2^Department of Military and Emergency Medicine, Uniformed Services University of the Health Sciences, Bethesda, Maryland; ^3^Heller Institute of Medical Research, Sheba Medical Center, Tel Hashomer, Israel; ^4^The Israel Defense Forces Institute of Military Physiology, Tel Hashomer, Israel; ^5^Sackler Faculty of Medicine, Tel Aviv University, Ramat Aviv, Israel; ^6^University of Otago, School of Physical Education, Sport and Exercise Sciences, Dunedin, New Zealand; ^7^Faculty of Health Sciences, University of Primorska, Polje, Izola, Slovenia

**Keywords:** core body temperature, heat injury, individualized mathematical model, Kalman filter, noninvasive measurements

## Abstract

A rising core body temperature (T_c_) during strenuous physical activity is a leading indicator of heat-injury risk. Hence, a system that can estimate T_c_ in real time and provide early warning of an impending temperature rise may enable proactive interventions to reduce the risk of heat injuries. However, real-time field assessment of T_c_ requires impractical invasive technologies. To address this problem, we developed a mathematical model that describes the relationships between T_c_ and noninvasive measurements of an individual’s physical activity, heart rate, and skin temperature, and two environmental variables (ambient temperature and relative humidity). A Kalman filter adapts the model parameters to each individual and provides real-time personalized T_c_ estimates. Using data from three distinct studies, comprising 166 subjects who performed treadmill and cycle ergometer tasks under different experimental conditions, we assessed model performance via the root mean squared error (RMSE). The individualized model yielded an overall average RMSE of 0.33 (SD = 0.18)°C, allowing us to reach the same conclusions in each study as those obtained using the T_c_ measurements. Furthermore, for 22 unique subjects whose T_c_ exceeded 38.5°C, a potential lower T_c_ limit of clinical relevance, the average RMSE decreased to 0.25 (SD = 0.20)°C. Importantly, these results remained robust in the presence of simulated real-world operational conditions, yielding no more than 16% worse RMSEs when measurements were missing (40%) or laden with added noise. Hence, the individualized model provides a practical means to develop an early warning system for reducing heat-injury risk.

**NEW & NOTEWORTHY** A model that uses an individual’s noninvasive measurements and environmental variables can continually “learn” the individual’s heat-stress response by automatically adapting the model parameters on the fly to provide real-time individualized core body temperature estimates. This individualized model can replace impractical invasive sensors, serving as a practical and effective surrogate for core temperature monitoring.

## INTRODUCTION

Athletes, Armed Forces personnel, and industrial workers are at risk for heat illness when they perform intense physical activities in hot and humid conditions. Such exertional heat illness is the third leading cause of sudden death in sport, with rates of incidence on the rise among sport, military, and industrial populations (3, 9, 25, 26, 52). Every year, the United States (U.S.) military consistently reports ~2,000 cases of heat injuries despite a continued focus on prevention (3), and over 9,200 American high school students are treated for exertional heat illness (9, 52). Meanwhile, heat injuries cause 33 yearly deaths among U.S. industrial populations (25). During strenuous, goal-oriented physical activities, such as military operations or athletic competitions, humans may either overlook or fail to perceive subtle thermoregulatory changes that can lead to heat injuries (16).

An unregulated rise in core body temperature (T_c_) is a leading indicator of heat-injury risk. Under normal conditions, the human thermoregulatory system maintains homeostasis at a T_c_ around 37°C. During compensable exercise, T_c_ can rise by a few degrees and return back to its homeostatic level postexercise (i.e., a regulated rise). However, during strenuous physical activity in hot and humid conditions, the thermoregulatory system may be unable to cope with the rate of heat production and thus fail to curb a rising T_c_. This could trigger a cascade of clinical responses, starting with mild degradation of physical and cognitive performance that progresses to heat exhaustion and then heat stroke and culminates in multiorgan dysfunction and potentially death (14, 16, 24, 45, 57).

A system that accurately measures T_c_, reliably predicts the onset of T_c_ increase, and generates early warnings may enable proactive interventions that could potentially prevent or reduce the risk of heat injuries. However, obtaining invasive medical-grade (i.e., gold-standard) T_c_ measurements from the pulmonary artery is impractical in ambulatory settings (e.g., during military field training and athletic activities). For exercise monitoring, especially in indoor laboratory settings, rectal temperature is the accepted gold-standard measure of T_c_ (8, 23, 29, 40). However, the invasiveness of the temperature sensor and the relative discomfort it can cause for long-duration monitoring make rectal probes impractical for use in outdoor settings, such as those involving military training or field operations (13, 44). Ingestible thermometer pills, which in the last decade have been used successfully in field settings (38), are considered a reliable means to measure T_c_. However, their cost and the practical difficulties in continually monitoring a large number of subjects for long-duration activities make them an unattractive option. Noninvasive methods to measure T_c_ via axillary or tympanic temperatures have not performed as well as gold-standard rectal measurements during exercise, as reported by several previous studies (8, 23, 29, 40, 44). However, more recent studies suggest that, while they can provide adequate measurements, their accuracy depends on sensor location and type of tympanic device. These studies also argue for extensive validation before field use (21, 58).

Recent advances in commercially available wearable devices (e.g., fitness-tracking wristwatches), which provide increasingly reliable noninvasive measurements of physiological variables, such as heart rate (HR), skin temperature (T_s_), and physical activity (A_c_, as measured by a 3-axis accelerometer), allow for the development of computational algorithms that combine these data through mathematical models to provide individualized T_c_ estimates in real time. Two modeling approaches have been proposed. The first approach relies on using first-principles models that describe heat production in the body in response to physical activity, heat transfer from the body core to the skin, and that from the skin to the environment via a series of macroscopic heat-balance equations (17–19, 22). However, although some of these models are very detailed, they invariably include a large number of parameters, which presents challenges for adapting the model to an individual under different conditions. The second approach relies on data-driven models that use nonlinear functions derived from population-average data to relate noninvasive measurements of physiological variables to T_c_ (5, 49). Although promising, these models do not account for the large interindividual variability in response to heat stress [e.g., for acclimated vs. nonacclimated individuals (14)], especially at high T_c_ values, which are most relevant for heat injuries but for which data to train such models are scarce (49). A parsimonious mathematical model that includes a limited number of parameters that can be adapted on the fly to learn an individual’s heat-stress response could potentially overcome these challenges.

To achieve this goal, we first formulated a mathematical model that describes the relationships between T_c_ and noninvasive measurements of A_c_, two physiological signals (HR and T_s_), and two environmental variables [ambient temperature (T_a_) and relative humidity (RH)]. We then hypothesized that this model would yield accurate personalized T_c_ estimates if its parameters were continually adapted to each individual. To this end, we coupled the mathematical model to a Kalman filter (32), which automatically adapts the model parameters to each individual on the fly in real time, in response to the individual’s physical activity and, in doing so, accounts for the individual’s sex, fitness, hydration status, exercise intensity, acclimatization level, clothing, and environmental condition. This customizability of the model is based on the premise that physiological variables (HR and T_s_) measured from an individual are reflective of subject-specific differences in the factors mentioned above. To evaluate the performance of the individualized model, we simulated real-time operation by allowing the model to automatically learn the physiological heat-stress response of 166 subjects exposed to different exertional and environmental conditions in three separate laboratory studies and then directly compared the estimated T_c_ against the corresponding measurements. Finally, because we eventually intend to use the model-estimated T_c_ as a surrogate for core temperature measurements, we performed the following two additional analyses: *1*) we verified whether the model-estimated T_c_ allowed us to reach the same findings as those obtained in the three studies using the measured T_c_ data, and *2*) we tested whether the model was robust to real-world operational conditions, such as nonavailable or unreliable measurements, by simulating these scenarios and evaluating the accuracy of the model-estimated individualized T_c_ profiles.

## METHODS

We used data from three distinct previously reported studies, comprising 166 subjects who performed treadmill and cycle ergometer tasks under different experimental and environmental conditions. Table 1 provides the demographic information for each of the three studies described below.

**Table 1.** Demographic characteristics of the subjects in our three studies

Study No.	No. of Subjects	Sex	Age, yr	Height, m	Weight, kg	BMI, kg/m^2^	Ref. No.
*1*	60	42 Men	27 (6)	1.79 (0.07)	85.23 (10.70)	26.67 (2.81)	41
		18 Women	27 (5)	1.66 (0.05)	64.64 (7.12)	23.64 (2.29)	
*2*	96	Men	20 (1)	1.76 (0.06)	74.57 (11.71)	24.72 (4.66)	12a, 34
*3*	10	Men	31 (8)	1.76 (0.06)	80.05 (11.99)	25.94 (4.60)	46

Data are mean values with SD in parentheses. BMI, body mass index.

### 

#### Study 1.

Sixty subjects (42 men and 18 women) were recruited from military and university communities to participate in a standardized heat-tolerance test (HTT) (41). The HTT is widely used in the military community to assess the ability of an individual to return to duty after previously suffering a heat injury (43). It involves a 2-h walk at 5 km/h on a treadmill set to a 2% grade in an environmental chamber set to T_a_ = 40°C and RH = 40%. In the context of the HTT, an individual is deemed heat intolerant if T_c_ exceeds 38.5°C and HR exceeds 150 beats/min, or when neither tends to plateau by the end of the protocol. The Israeli Defense Force has been using the HTT for over 30 yr to help screen military personnel for return to active duty (12a). The Institutional Review Board of the Uniformed Services University (Bethesda, MD) approved the study. Each subject, after being briefed on the purposes and procedures of the study, gave written informed consent before study participation.

During the study, each subject reported to the Uniformed Services University’s environmental chamber on two occasions. Upon arriving in the morning, the subject changed into shorts and athletic shoes (women additionally wore a sports bra). Subjects underwent the standard HTT on one testing day. On another testing day, subjects underwent an identical test except with a T_a_ of 22°C (the order was randomized). After walking, subjects rested and cooled down for at least 20 min (or until T_c_ <38.0°C) while physiological measurements continued. The average time between the two testing days was 4 days. During the HTT, subjects could drink water ad libitum (up to 1 l/h). T_c_ was measured by a rectal thermometer inserted 10 cm beyond the anal sphincter (MEAS Temperature Probe; Measurement Specialties, Dayton, OH), T_s_ was measured by a skin sensor at the chest (YSI 409B; YSI, Yellow Springs, OH), and HR was measured by a Polar HR monitor (Polar Team^2^ Pro; Polar, Lake Success, NY). HR was monitored and recorded every second while T_c_ and T_s_ were recorded throughout the test at 15-s intervals (41). For maintaining a consistent sampling period across the data sets from the three studies, we resampled the HR and T_s_ signals to 1-min intervals by averaging the data. For our mathematical model, we identified the metabolic equivalent unit (MET: the ratio of oxygen consumed during a specific physical activity to that at rest) for a walking speed of 5 km/h from the Compendium of Physical Activities (1) and then inferred the A_c_ level to be moderate (MET ~3–6), following a previous study (51).

#### Study 2.

Similar to *study 1*, 96 subjects (all men) performed the standard HTT protocol (walking on a treadmill set to a 2% grade at 5.0 km/h for 120 min) at a T_a_ of 40°C and a RH of 40%. The Institutional Review Board of the Israel Defense Forces Medical Corps approved the use of the data for retrospective studies (12a, 34).

Throughout the duration of the walk, T_c_ was measured by a rectal thermometer inserted 10 cm beyond the anal sphincter (YSI 401; YSI), HR was measured by a Polar HR monitor (Polar Team^2^ Pro; Polar) and stored at 1-min intervals by a heart watch (RS800cx watch; Polar Electro Oy, Kempele, Finland), and T_s_ was measured by a skin sensor at the chest (YSI 409B; YSI). Temperature data were continually monitored and recorded throughout the test at 1-min intervals. Similar to *study 1*, we inferred the A_c_ level to be moderate for a walking speed of 5 km/h.

#### Study 3.

Ten active and healthy men participated in this study, which was approved by the University of Otago’s Human Ethics Committee in accordance with the Declaration of Helsinki. Subjects were given a concise explanation of all experimental procedures and potential risks. Subsequently, they gave their written informed consent (46).

The participants, who were local cyclists ranging from recreationally active individuals to regional multisport athletes, were not heat acclimated. Subjects completed the following four trials in counterbalanced order after random assignment: *1*) precooling before exercise and no fan airflow during exercise (PCNF), *2*) no precooling and no fan airflow (NCNF), *3*) precooling with fan airflow (PCWF), and *4*) no precooling with fan airflow (NCWF). Of the 10 subjects, the requisite data (HR, T_s_, and T_c_ measurements) were available for all four trials for four subjects and for at least two of the four trials for the remaining six subjects. Each subject performed the trials at the same time of day, with trials separated by at least 7 days.

The test protocol started with the subject laying submerged chest deep in a custom-insulated bath for 1 h before exercising, in either thermoneutral water (35°C) or cool water (24°C; precooling was stopped when 1 h had elapsed or when T_c_ decreased by 0.5°C, whichever occurred earlier). Each subject began the cycling protocol on an electromagnetically braked cycle ergometer (Velotron version 1.5; RaceMate, Seattle, WA) exactly 10 min after exiting the bath; this period was required for drying off, changing, and relocating to a temperature-controlled environmental chamber (T_a_ = 30°C; RH = 50%). During trials requiring airflow, a large fan (655-mm-diameter blade; Imasu IMS International, Tsuen Wan, Hong Kong) was placed 1 meter in front of the subject. The fan height was adjusted to include airflow over the head, torso, arms, and upper legs, covering as much surface area as possible in the cycling position; the maximum average wind velocity at 1 meter was 4.8 m/s.

During the cycling trials, the air exhaled by the subject was sampled breath by breath to calculate the rates of ventilation, oxygen uptake, and CO_2_ production (Cortex Biophysik Metalyzer 3B, Leipzig, Germany). For our mathematical model, we computed METs from the rates of oxygen uptake and converted these to A_c_ levels (51). HR was monitored via a chest-strap device (Vantage NV; Polar Electro, Port Washington, NY). T_s_ was measured at 10 sites on the right side of the body via insulated skin thermistors affixed to the skin surface with adhesive tape (Type EU; Grant Instruments, Cambridge, UK), and the measurements were used to compute a mean value. T_c_ was monitored via an esophageal thermistor while the subject was in the bath and via a rectal thermistor placed 10 cm past the anal sphincter while the subject exercised (Mon-a-therm 400; Mallinckrodt Medical, St. Louis, MO). The measured data (HR, T_c_, T_s_, oxygen uptake, and CO_2_ production) were logged at 1-min intervals (Grant 1200 series Squirrel data logger; Grant Instruments).

#### Individualized model.

**Fig. 1.**
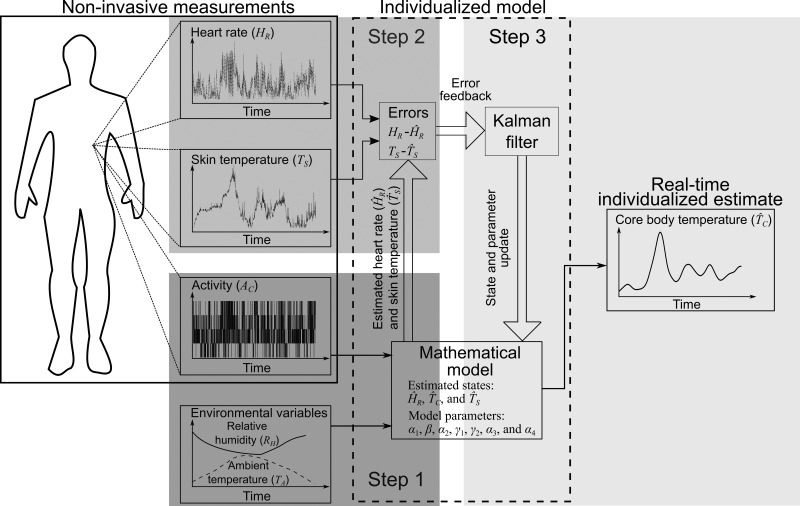
The proposed model for individualized core body temperature (T_c_) estimation. The inputs to the model are the measured heart rate (HR), skin temperature (T_s_), and physical activity (A_c_) profiles from an individual and two environmental variables [ambient temperature (T_a_) and relative humidity (RH)]. *Step 1* (dark gray), the mathematical model uses the activity profile and environmental variables to estimate the HR and T_s_. *Step 2* (medium gray), the system computes the errors between the model-estimated HR and T_s_ and the corresponding measurements. *Step 3* (light gray), the Kalman filter uses the errors to update six model parameters (α_1_, β, γ_1_, γ_2_, α_3_, and α_4_) and provide individualized real-time core temperature estimates. We fixed α_2_, the thermoregulatory rate constant of T_c_, to a constant value. Parameter definitions: α_1_, the rate constant of HR; β, the gain in HR in response to A_c_; γ_1_, the heat gain resulting from metabolic activity; γ_2_, the rate of heat transfer from the core to the skin compartment; α_3_, the rate of heat transfer from the skin to the environment via convection; and α_4_, the rate of heat transfer resulting from sweat evaporation.

The proposed model uses an individual’s noninvasive measurements of A_c_, HR, and T_s_, as well as two environmental variables, T_a_ and RH, to estimate the individual’s T_c_ in real time. The model includes two elements, a mathematical model and a Kalman filter (32), which together provide real-time individualized estimates of T_c_ via the following three steps (Fig. 1): *1*) first, in *step 1*, the mathematical model uses the measured (or computed) activity A_c_ and environmental variables T_a_ and RH as inputs to estimate values of HR and T_s_ (i.e., to compute the state variables $\hat{HR}$ and T̂_s_); *2*) next, in *step 2*, the system computes the errors between the measured HR and T_s_ and the estimated values $\hat{HR}$ and T̂_s_; and *3*) finally, in *step 3*, to reflect the individual’s physiological response, the Kalman filter considers these errors to correct the state variables and update the mathematical model parameters, which are then used in the subject-adapted model to provide T̂_c_, an improved estimate of the individual’s T_c_. We refer to the combination of the mathematical model and the Kalman filter as the “individualized” model. We have provided details of the Kalman filter algorithm in appendix a.

#### Mathematical model.

The mathematical model consists of the following two submodels: a phenomenological component that relates A_c_ to HR and a first-principles macroscopic energy-balance component (17–19) that relates metabolic activity (represented by HR) to T_s_ and T_c_.

The structure of the phenomenological model was motivated by the observation that an increase in A_c_ leads to a rapid increase in HR, which subsequently decays exponentially when A_c_ decreases. This relationship was mathematically represented by the following equation:

(1) $$\frac{\text{dΔHR}}{\text{d}t}=-\alpha_{1}\Delta HR+\text{β}A_{\text{c}}^{4},$$

where ΔHR denotes the change in HR from a resting state HR_0_ (i.e., ΔHR = HR *–* HR_0_), α_1_ denotes the rate constant for HR, and β represents the gain in HR resulting from physical activity. Here, we set HR_0_ as the mean of the measured HR during the initial 10 min of data (~80 beats/min) obtained under light activity levels. Following a previous study (51), we quantized human activities into specific MET values, which were further classified into five activity levels as follows: A_c_ = 0 for rest (MET = 1), A_c_ = 1 for light activity (MET ~1–3), A_c_ = 2 for moderate activity (MET ~3–6), A_c_ = 3 for high activity (MET ~6–9), and A_c_ = 4 for very high activity (MET ≥ 9). In *Eq. 1*, we raised A_c_ to the fourth power to ensure good separation of HR at different activity levels because we noticed during data analysis that moderate activity (A_c_ = 2) leads to a greater increase in HR compared with light activity (A_c_ = 1).

The first-principles component of the model consists of the core- and skin-temperature compartments:

(2) $$\frac{\text{dΔTc}}{\text{d}t}=-\alpha_{2}\Delta Tc+\gamma_{1}S\text{(ΔHR)ΔHR}-\gamma_{2}(Tc-TS),$$

(3) $$\frac{\text{dΔTs}}{\text{d}t}=-\alpha_{3}(Ts-Ta)-\alpha_{4}(Ps-\text{Pa)}+\gamma_{2}(Tc-\text{Ts}),$$

where ΔT_c_ = T_c_ – T_c0_ and ΔT_s_ = T_s_ – T_s0_, with T_c0_ and T_s0_ denoting the initial core and skin temperatures, respectively, P_s_ denotes the vapor pressure of water for T_s_, and P_a_ represents the vapor pressure of water at a given T_a_ and RH, as calculated by the perceived heat index. Here, we set T_c0_ to 37°C and T_s0_ as the mean of the measured T_s_ during the initial 10 min of data collection. We used the equation proposed by Rothfusz (50) to compute the heat index (for a given T_a_ and RH) and computed the vapor pressure of water via the Antoine equation (56): log_10_(P) = *A –* [*B*/(*C +* T)], where *A* = 8.07, *B* = 1730.63, and *C* = 233.43, with T = T_s_ for P *=* P_s_ and T = heat index for P *=* P_a_ (both in units of °C). In *Eq. 2*, α_2_ denotes the thermoregulatory rate constant of T_c_, γ_1_ denotes the rate of heat gain due to metabolic activity (HR), *S*(ΔHR) is the standard sigmoid function to reduce the heat gain to zero when HR decreases below the resting state (i.e., when ΔHR becomes negative), and γ_2_ denotes the rate of heat transfer from the core to the skin compartment. In *Eq. 3*, α_3_ denotes the rate of convective heat transfer from the skin compartment to the environment, and α_4_ denotes the rate of heat loss to the environment due to sweat evaporation. Thus, the mathematical model consists of three states (ΔHR, ΔT_c_, and ΔT_s_, corresponding to *Eqs. 1*, *2*, and *3*, respectively) and seven parameters (α_1_, β, α_2_, γ_1_, γ_2_, α_3_, and α_4_).

#### Initial values of model parameters.

We used published data from four studies to estimate the parameters α_1_ and β, which relate A_c_ to HR via *Eq. 1* (4, 20, 37, 53). The studies involved a total of 184 subjects performing treadmill runs or cycle ergometer tasks at different speeds, with A_c_ ranging from zero (rest) to four (very high). We obtained the physiological ranges of α_1_ and β by performing a least-squares fit of *Eq. 1* to the data from these studies. We fixed α_2_ at a constant value because it represents the rate of self-regulated changes in T_c_, which is invariant across individuals (28, 36). We obtained the physiological ranges of the other four parameters (γ_1_, γ_2_, α_3_, and α_4_) from published values of human tissue and heat transfer properties from experimental studies and the human numbers database (2, 18, 27, 30, 31, 48, 54). We computed the means and SDs of the six parameters from the physiological ranges thus obtained and used these values to initialize the individualized model.

#### Evaluation of model performance.

We evaluated the ability of the individualized model to learn a subject’s heat-stress response under different environmental and experimental conditions by simulating real-time estimates of each subject’s T_c_ (i.e., T̂_c_) and comparing them against the corresponding experimental measurements. To assess the accuracy of T̂_c_, we computed the square root of the mean squared differences between T̂_c_ and the gold-standard measurements of T_c_ [i.e., the root mean squared error (RMSE)] throughout the duration of the experiment for each of the 166 subjects under each experimental condition. Note that T_c_ measurements are only used in this study to assess the model estimates T̂_c_. In addition, to assess the accuracy of the model for T_c_ values that are elevated enough to be clinically meaningful while still being able to analyze a reasonable fraction of the data, we computed the RMSE between T̂_c_ and T_c_ for measurements >38.5°C. Across the three studies, 22 unique subjects had T_c_ values exceeding 38.5°C in at least one of the experimental conditions (34 individual profiles in total). Previous studies suggest that a rising T_c_ may lead to a cascade of responses, starting with degradation of aerobic performance and reduced cognitive function at around 38.2°C and beyond (14, 24, 45) that progresses to heat exhaustion (33, 42, 57) and to heat stroke (16, 57). Importantly, there is no specific T_c_ threshold that delineates the transition from one state to the next. Rather, a subject’s response to an increasing T_c_ depends on several factors, which include the subject’s hydration status, clothing, environmental condition, exercise intensity, fitness, and core-to-skin temperature gradient (12, 15, 33, 42). Hence, here we used 38.5°C as a tradeoff between a low-enough value to support the analyses of a reasonable-size data set (22 subjects in 34 profiles) and a high-enough value that is clinically meaningful.

## RESULTS

### 

#### Evaluation of model performance.

Table 2 shows the average RMSE at all T_c_ values and the average RMSE for T_c_ >38.5°C, across each experimental condition for the three studies. The individualized model (Table 2, model with T_s_) yielded an average error of 0.33 (SD = 0.18)°C. Importantly, for the 22 subjects whose T_c_ exceeded 38.5°C (34 individual profiles; in some cases, the same subject exceeded the threshold in two or more experimental conditions), the average error decreased to 0.25 (SD = 0.20)°C. In *study 3*, there were two subjects whose T_c_ exceeded 39°C in three or more experimental conditions. For these subjects (*3* and *7*, seven time profiles), the model yielded an average RMSE of 0.22 (SD = 0.03)°C for T_c_ >39.0°C. In addition, we assessed the individualized model’s ability to reproduce the magnitude of the measured peak T_c_ (max T_c_, see appendix b for details) and found that, on average, the absolute difference between max T_c_ and T̂_c_ at the same time point was 0.27 (SD = 0.20)°C across all subjects in the three studies.

**Table 2.** Performance of the individualized model in estimating T_c_ of subjects from the three studies with and without T_s_ included as input to the model

				Model with T_s_				Model without T_s_			
				For all T_c_		T_c_ >38.5°C		For all T_c_		T_c_ >38.5°C	
Study (No. of Subjects)	Condition	No. of Profiles per Condition	No. of Profiles with T_c_ >38.5°C	True HR	Noisy HR	True HR	Noisy HR	True HR	Noisy HR	True HR	Noisy HR
1 (60)*	22°C	60		0.32 (0.15)	0.34 (0.14)			0.32 (0.16)	0.35 (0.16)		
	40°C	60	2	0.35 (0.16)	0.34 (0.14)	0.14 (0.05)	0.15 (0.05)	0.34 (0.17)	0.35 (0.18)	0.18 (0.00)	0.17 (0.01)
2 (96)*	40°C	96	14	0.30 (0.14)	0.30 (0.13)	0.24 (0.19)	0.28 (0.18)	0.31 (0.15)	0.31 (0.14)	0.22 (0.16)	0.27 (0.14)
3 (10)^†^	NCNF	7	3	0.40 (0.20)	0.38 (0.17)	0.29 (0.15)	0.29 (0.06)	0.36 (0.17)	0.35 (0.14)	0.27 (0.08)	0.30 (0.04)
	PCNF	10	5	0.56 (0.34)	0.56 (0.32)	0.26 (0.27)	0.28 (0.28)	0.55 (0.33)	0.54 (0.31)	0.39 (0.20)	0.40 (0.22)
	NCWF	7	6	0.33 (0.21)	0.33 (0.17)	0.30 (0.26)	0.35 (0.24)	0.31 (0.20)	0.32 (0.15)	0.21 (0.16)	0.27 (0.15)
	PCWF	7	4	0.47 (0.27)	0.48 (0.23)	0.21 (0.20)	0.32 (0.18)	0.46 (0.27)	0.46 (0.24)	0.25 (0.33)	0.31 (0.29)
Total (166)		247	34	0.33 (0.18)	0.33 (0.16)	0.25 (0.20)	0.29 (0.19)	0.33 (0.18)	0.33 (0.17)	0.25 (0.18)	0.29 (0.17)

For the two models, entries indicate mean root mean squared error (RMSE) values with SD in parentheses. RMSE (°C) is shown throughout the duration of the experiment and during episodes where core body temperature (T_c_) > 8.5°C. Columns labeled Noisy HR show the effects of adding noise to the HR measurements on model performance. T_s_, skin temperature; HR, heart rate; NCNF, no precooling and no fan airflow during exercise; NCWF, no precooling but with fan airflow during exercise; PCNF, precooling and no fan airflow during exercise; PCWF, precooling but with fan airflow during exercise.

* *Study 1* was conducted under two different ambient temperature conditions, whereas *study 2* was conducted at 40°C. In both studies, the relative humidity was set to 40%.

† *Study 3* was conducted at an ambient temperature of 30°C at a relative humidity of 50%.

#### Ability of the individualized model to learn the thermoregulatory response of the same subject under different experimental conditions.

**Fig. 2.**
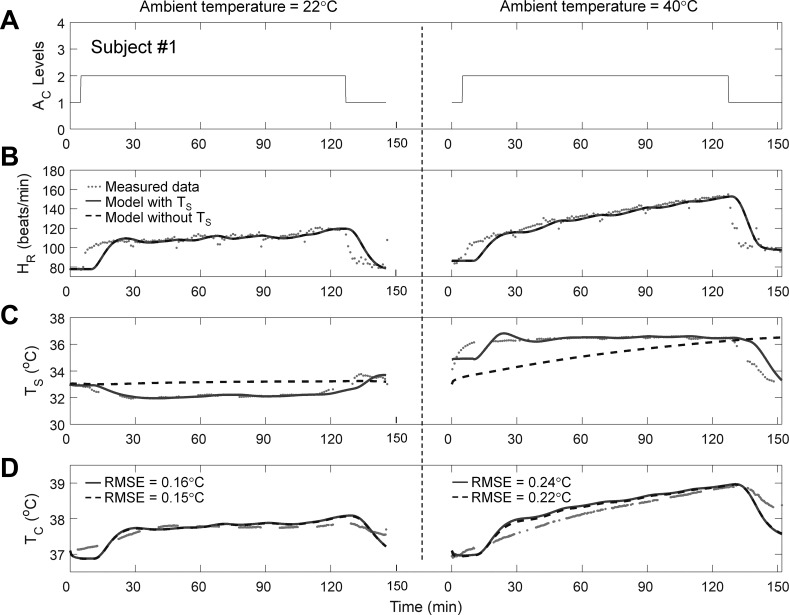
Performance of the individualized model in predicting the T_c_ of one subject from *study 1*. *A*: activity profiles of the subject during heat tolerance tests at two T_a_ levels at a RH of 40%. *B*–*D*: measured and estimated values of HR, T_s_, and T_c_, respectively, including the root mean squared error (RMSE) for the T_c_ estimates. Gaps in T_s_ and T_c_ data (*C* and *D*, respectively) are because of missing measurements.

A novel feature of the individualized model is its ability to adapt the model parameters to an individual subject and, as such, explicitly account for subject-specific variations in thermoregulatory responses under different environmental or experimental conditions. Figure 2 shows an example of the model’s ability to estimate a subject’s T_c_ (*study 1*, *subject 1*) for the same activity (treadmill task) performed at two different T_a_ values, 22°C and 40°C. Figure 2*A* shows the measured A_c_ levels that drove the individualized model to track the measured HR and T_s_ (Fig. 2, *B* and *C*, respectively) via the Kalman filter and provides real-time T_c_ estimates (Fig. 2*D*). Changes in $\hat{HR}$ lagged behind those in HR, both at the beginning and end of the moderate activity (A_c_ = 2) period (up to ~15 min and after ~125 min; Fig. 2*B*). At the beginning, the delay occurred because of model initialization (the initial 10-min period during which HR was already increasing). This was subsequently followed by a small delay (~5 min) introduced by the causal noise-rejecting filter applied to HR before the Kalman filter algorithm became engaged (see appendix a). At the end of the moderate activity period, the noise-rejecting filter alone caused the delay in $\hat{HR}$. As expected, the T_c_ values in the 40°C condition were higher than those in the 22°C condition, an effect that the model captured. The individualized model yielded errors of 0.16°C and 0.24°C for the 22°C and 40°C conditions, respectively.

**Fig. 3.**
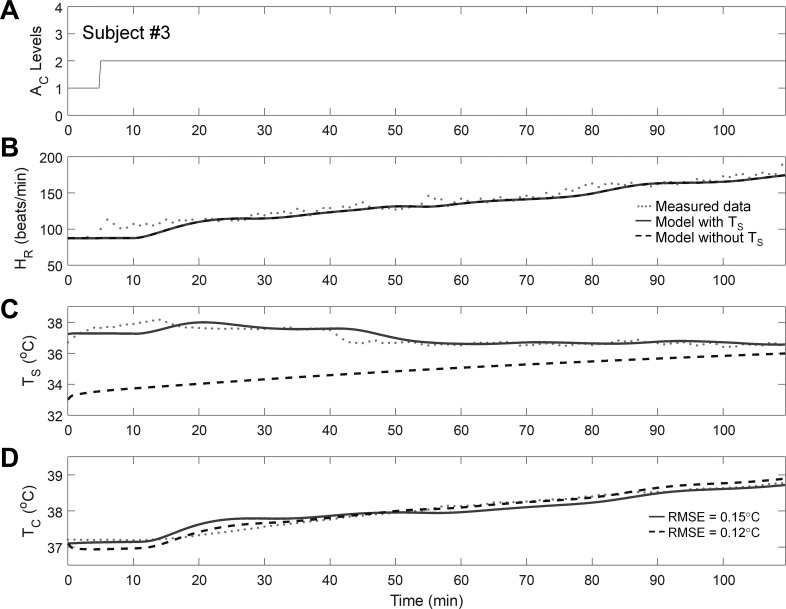
Performance of the individualized model in predicting the T_c_ of one subject from *study 2*. *A*: activity profile during a heat tolerance test at an T_a_ of 40°C and a RH of 40%. *B*–*D*: measured and estimated values of HR, T_s_, and T_c_, respectively, including the RMSE for the T_c_ estimates.

Figure 3 shows the performance of the model in predicting the T_c_ of one subject from *study 2* (*subject 3*) at T_a_ = 40°C and RH = 40%. As in *study 1*, despite a minor lag in $\hat{HR}$ in the beginning (the initial 15 min), the model generally captured the rise in T_c_ for the 14 subjects whose T_c_ exceeded 38.5°C in *study 2* [average error = 0.24 (SD = 0.19)°C; Table 2].

**Fig. 4.**
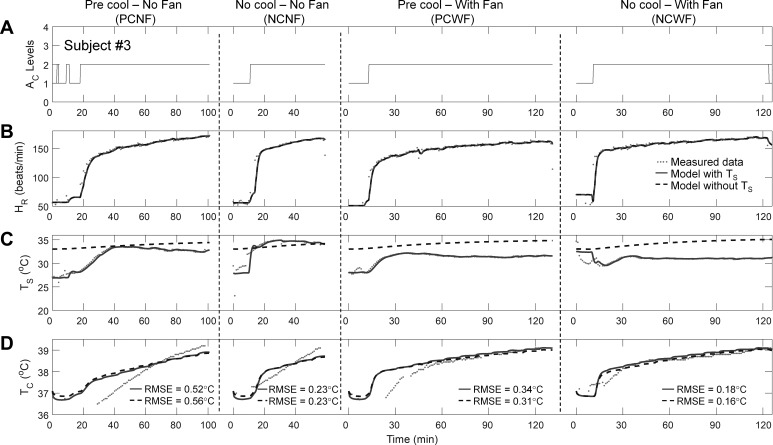
Performance of the individualized model in predicting the T_c_ of one subject from *study 3*. *A*: activity profiles of the subject while performing the cycle task under four experimental conditions at an T_a_ of 30°C and a RH of 50%. *B*–*D*: measured and estimated values of HR, T_s_, and T_c_, respectively, including the RMSE for the T_c_ estimates.

Figure 4 shows an example of the model’s ability to estimate a subject’s T_c_ (*study 3*, *subject 3*) for the same activity (cycle ergometer task) performed under four different experimental conditions in an environmental chamber maintained at a T_a_ of 30°C and a RH of 50%. In the precooling conditions (PCNF and PCWF), the model overestimated T_c_ up to 60 min into the task (Fig. 4*D*). This discrepancy reflects the observation that, whereas HR increased rapidly (by ~70 beats/min in 10 min), rectal T_c_ measurements increased more slowly. In these conditions, the rectal measurements may have lagged (35, 40) and underestimated the true T_c_ because of the influence of the volume of cutaneous blood cooled by water (or other cooling device) circulating in the veins of the legs during cycling (39). Nonetheless, at later time points when T_c_ values were high, which are important in predicting the risk of heat injuries, the model showed good accuracy across the four experimental conditions.

#### Adaptation of model parameters.

**Fig. 5.**
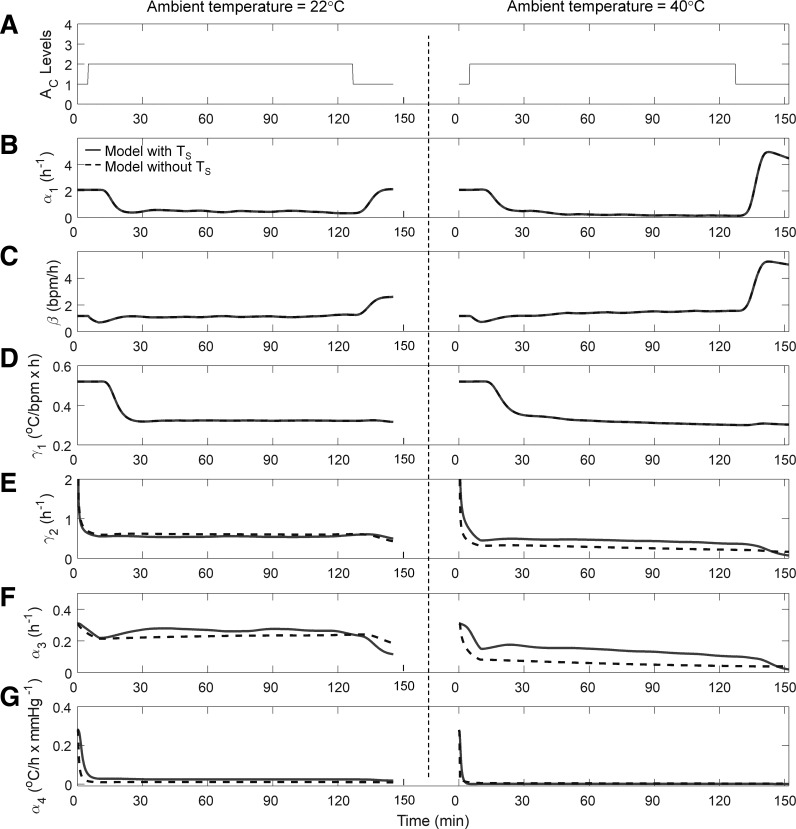
Time profiles of the six adjustable model parameters (α_1_, β, γ_1_, γ_2_, α_3_, and α_4_) in the individualized model for the same subject from *study 1* shown in Fig. 2. *A*: activity profiles of the subject during heat tolerance tests at two T_a_ levels at a RH of 40%. *B*–*G*: time profiles of the six parameters.

To describe how the model adapts to different heat-stress conditions in the same individual, we examined how the adjustable parameters changed as a function of time. Figure 5 shows the dynamical changes in the six adjustable parameters of the individualized model as it adapted to the subject in Fig. 2 under two conditions (T_a_ = 22 and 40°C).

Before interpreting the figure, we note that not all model parameters are equally sensitive to changes in T_a_. Parameters α_1_ and β (*Eq. 1*), which denote the rate constant of and gain in HR, respectively, are affected by changes in HR due to A_c_ and T_a_. Parameters γ_1_ and γ_2_ (*Eq. 2*), which denote the rates of heat gain resulting from metabolic activity and heat transfer to the skin compartment, respectively, are affected by changes in T_s_ due to T_a_. However, the effects of T_a_ on T_c_ via these two parameters (γ_1_ and γ_2_) are minimal because the skin and deeper tissues effectively insulate the body core from variations in environmental conditions (2, 16). The parameter α_3_ (*Eq. 3*), which denotes the rate of heat loss from the skin to the environment via convection, is the only parameter that is directly affected by changes in T_a_ and hence is the most sensitive. We also note that, depending on the condition, the model parameters require between 30 and 45 min to converge and adapt to an individual’s response to heat stress.

Upon initiation of the HTT, A_c_ changed from light to moderate (i.e., it changed from 1 to 2; Fig. 5*A*), causing the HR to rise (Fig. 2*B*). In both temperature conditions, α_1_, the rate constant of the HR, initially decreased up to ~25 min into the simulation to a considerably lower value than the initial value (Fig. 5*B*). Subsequently, α_1_ settled at this value for the 22°C condition (where the HR settled to ~110 beats/min) while continuing to decrease further in the 40°C condition (where the HR continued to increase up to ~150 beats/min). In both conditions, α_1_ constrained the rate of change in $\hat{HR}$ as it increased (Fig. 2*B*). Parameter β, the gain in HR due to A_c_, decreased initially because $\hat{HR}$ remained constant during the initial 10 min of model initialization. Subsequently, β increased nominally (Fig. 5*C*), contributing to the rise in HR. The sharp increases in α_1_ and β at the end of the moderate activity (A_c_ = 2) period contributed to the decrease in the HR (after ~135 min). Thus, these two parameters are highly sensitive to A_c_-induced variations in HR.

Parameter γ_1_, the rate of heat gain because of metabolic activity, decreased as T_c_ increased, effectively dampening the rise of T_c_ in response to a rise in HR (Fig. 5*D*). Parameter γ_2_, the rate of heat transfer to the skin compartment, varied as a function of the difference between T_s_ and T_c_; it rapidly decreased as T_c_ increased, whereas T_s_ remained almost constant in both conditions, because heat was not dissipated to the skin (Figs. 2, *C* and *D*, and 5*E*).

Parameter α_3_, the rate of heat loss from the skin to the environment via convection, decreased more at 40°C than at 22°C (Fig. 5*F*). This was expected because, at T_a_ = 22°C, the difference between T_s_ and T_a_ was large (~10°C), increasing convective heat loss. In contrast, at T_a_ = 40°C, the gradient was small (~4°C), effectively reducing convective heat loss compared with the 22°C condition (Fig. 2*C*). Parameter α_4_, the rate of evaporative heat loss from the skin to the environment, decreased rapidly (within ~10 min) and remained low in both conditions (Fig. 5*G*). This was expected because α_4_ typically remains low unless there is considerable wetting of the skin and the environment is conducive to sweat evaporation.

#### Individualized model estimates corroborate study findings.

Apart from evaluating model performance (detailed in the sections above), we assessed whether T̂_c_ would lead to the same conclusions as those obtained from the measured T_c_ in each of the three studies.

The two major findings of *study 1* (41) were that *1*) a subject’s heat tolerance can only be assessed at high T_a_ levels (T_a_ = 40°C) and *2*) heat-intolerant subjects have a significantly higher T_c_ than do heat-tolerant subjects at a T_a_ of 40°C. To evaluate their findings, the authors computed two metrics from the measured data: max T_c_ and ΔT_c_ (the difference between max T_c_ and the T_c_ value achieved 1 h after commencing the treadmill task). Both metrics were lower at 22°C than at 40°C (*finding 1*, Tables 3–5). Furthermore, both metrics were lower for heat-tolerant subjects than for heat-intolerant subjects at 40°C (*finding 2*, Tables 3–5). Importantly, the same findings were obtained when T̂_c_ (Tables 3–5), instead of the measured T_c_, was used to compute the two metrics.

**Table 3.** Comparison of the measured T_c_ and the corresponding model estimate relating to the major findings in study 1

	T_a_ = 22°C		T_a_ = 40°C	
Measures	Data	Model	Data	Model
max T_c_, °C	37.66 (0.31)	37.74 (0.21)	38.02 (0.33)^†^	38.24 (0.31)^†^
ΔT_c_, °C	0.15 (0.16)	0.18 (0.11)	0.30 (0.17)^†^	0.26 (0.17)^†^

	Heat-tolerant subjects (T_a_ = 40°C)		Heat-intolerant subjects (T_a_ = 40°C)	
No. of subjects	46 (36 men, 10 women)		14 (6 men, 8 women)	
max T_c_, °C	37.94 (0.30)	38.15 (0.28)	38.28 (0.32)*	38.53 (0.22)*
ΔT_c_, °C	0.29 (0.16)	0.23 (0.15)	0.39 (0.21)*	0.36 (0.17)*

Entries indicate mean values with SD in parentheses. T_a_, ambient temperature; ΔT_c_, difference between the maximum T_c_ (max T_c_) and the T_c_ achieved after 1 h on the heat tolerance test (41).

* *P* < 0.05, Wilcoxon rank sum test comparing heat-tolerant vs. heat-intolerant subjects.

† *P* < 0.05, Wilcoxon signed-rank test comparing data (or model) values across the two different conditions.

**Table 5.** Comparison of the measured T_c_ and the corresponding model estimate relating to the major findings in study 3

	Time to T_c_ >38.5°C, min	
Condition	Data	Model
NCNF	32.20 (5.81)	34.40 (12.44)
PCNF	39.67 (14.08)	41.00 (13.37)
PCWF	39.00 (13.13)	49.50 (14.55)
NCWF	37.83 (5.81)	32.75 (5.80)

Entries indicate mean values with SD in parentheses.

The finding in *study 2* matched the second finding from *study 1*, that heat-intolerant subjects have significantly higher T_c_ than heat-tolerant subjects at a T_a_ of 40°C (12a, 34). Indeed, Tables 3–5 shows this finding to be true for both T_c_ and T̂_c_ (*study 2*).

The authors of *study 3* concluded that precooling slowed the rate of rise in T_c_ and increased exercise duration (46). We sought to test directly whether the individualized model could reproduce this effect by computing the time it takes for each subject’s T_c_ to exceed the heat-injury threshold of 38.5°C. Precooling increased the time to T_c_ >38.5°C [Tables 3–5, Data, NCNF vs. PCNF: 32.20 (SD = 5.81) min vs. 39.67 (SD = 14.08) min]. Although the results were not significant because of the small sample sizes across conditions, T̂_c_ corroborated the author’s conclusions [Tables 3–5, Model, NCNF vs. PCNF: 34.40 (SD = 12.44) min vs. 41.00 (SD = 13.37) min].

The authors of *study 3* also noted that this precooling effect occurred only in the absence of fan airflow. Indeed, the time to T_c_ >38.5°C for the PCWF was similar to the NCWF condition when using the measured T_c_ [Tables 3–5, Data, PCWF vs. NCWF: 39.00 (SD = 13.13) min vs. 37.83 (SD = 5.81) min]. For the model the times to T_c_ >38.5°C for the PCWF and NCWF conditions, despite diverging considerably, were not statistically different [PCWF vs. NCWF: 49.50 (SD = 14.55) min vs. 32.75 (SD = 5.80) min in Tables 3–5].

#### Robustness of individualized model.

To assess how the individualized model would perform if the wearable device could not measure T_s_, we evaluated a variant of the individualized model that disregarded the T_s_ measurements (model without T_s_; see appendix c for modifications to the Kalman filter algorithm). The performance of the model without T_s_ was comparable to that of the model with T_s_ for all subjects in each of the three studies [average RMSE = 0.33 (SD = 0.18)°C; average RMSE for the 22 subjects with T_c_ >38.5°C = 0.24 (SD = 0.18)°C; Table 2, also see Figs. 2–5].

To further investigate how the individualized model would estimate T_c_ when encountering operational conditions in real-world scenarios, we provided it with noise-laden or partially missing HR measurements and evaluated its performance (Table 2). To assess performance for noise-laden HR measurements, we used the procedure detailed in appendix d to simulate 100 random realizations by adding noise to the original HR data for each subject in each experimental condition. Briefly, to add realistic noise, we determined the error characteristics of the Samsung GearS3 smartwatch (Samsung Electronics America, Ridgefield Park, NJ), using 83 h of in-house HR measurements obtained while subjects performed different activities and comparing them against those obtained with a gold-standard chest-strap device (Polar H7; Polar Electro Oy). We then estimated the subject’s T_c_ for each of the 100 noise-laden HR profiles and computed 100 RMSE values by comparing the estimates against the measured T_c_ values. We then computed the average RMSE for that subject and repeated this procedure for each subject in each experimental condition across the three studies. Noise-laden HR data degraded model performance by only 16% for the 22 subjects whose T_c_ exceeded 38.5°C across the three studies [model with T_s_, true HR vs. noisy HR, RMSE for T_c_ >38.5°C: 0.25 (SD = 0.20)°C vs. 0.29 (SD = 0.19)°C; Table 2].

**Fig. 6.**
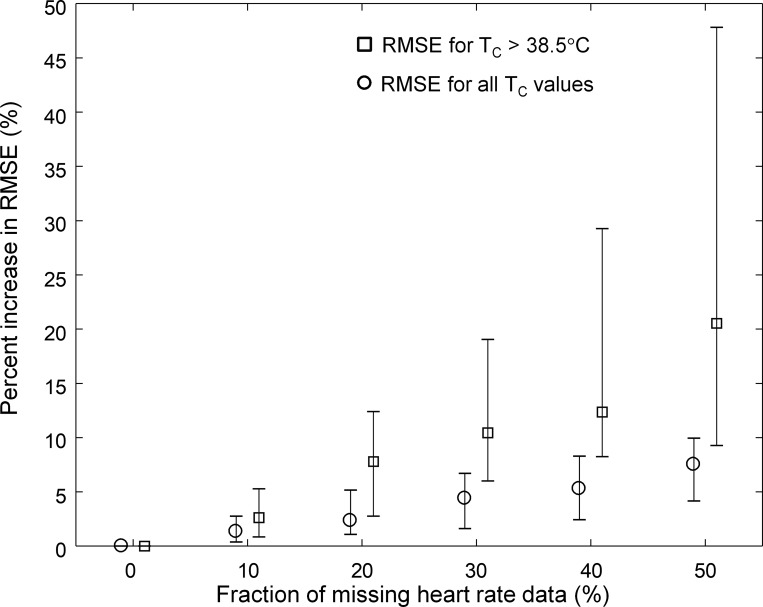
Effect of missing HR data on model performance. Using data from the 22 unique subjects (34 profiles from the 3 studies) whose T_c_ exceeded 38.5°C, we generated 100 random realizations of missing HR data for each fraction (from 10 to 50%) of the total number of HR samples for each of the 34 profiles. We computed the RMSE between the measured and estimated T_c_ values for each random realization and missing HR fraction in each of the 34 profiles. We then computed and plotted the percentage increase in the RMSE as a function of the fraction of missing HR data for each of the 34 profiles. Values are medians and interquartile ranges.

For missing HR data, we generated 100 random realizations for one subject in one experimental condition by randomly removing a fixed fraction of the total number of HR values. We varied the missing fraction from 10 to 50%, computed the average RMSE for each fraction of missing HR data, and computed the percentage increase in the RMSE relative to the RMSE obtained when using the true HR data (without missing values). We repeated this procedure for the 22 unique subjects (34 individual profiles across the different experimental conditions) whose T_c_ exceeded 38.5°C in any of the three studies. We then plotted the percentage increase in RMSE for all T_c_ values (Fig. 6) and the percentage increase in RMSE for T_c_ >38.5°C (Fig. 6) as a function of the percentage of missing HR data. Even with 40% of the data missing, the RMSE for T_c_ >38.5°C increased, on average, by only 13% (or 0.03°C).

## DISCUSSION

In this study, we developed an individualized model that uses activity data (A_c_) and noninvasive measurements of HR and T_s_, along with two environmental variables (T_a_ and RH), to provide real-time estimates of a subject’s T_c_. We demonstrated that the model estimated T_c_ with good accuracy across 166 subjects subjected to different exertional and environmental conditions [average RMSE = 0.33 (SD = 0.18)°C]. Furthermore, the estimation accuracy increased for T_c_ values exceeding 38.5°C [average RMSE = 0.25 (SD = 0.20)°C], a potential lower T_c_ limit of clinical relevance, when the ability to make accurate estimates starts to become important. We also showed that the model-estimated T_c_ (i.e., T̂_c_) corroborated the findings inferred from the corresponding measurements in each of the three studies, supporting the hypothesis that T̂_c_ could serve as a surrogate for invasive T_c_ measurements. Importantly, these results remained robust in the presence of simulated real-world operational conditions, such as nonavailability of T_s_ measurements (no difference in performance), random noise added to the HR data (16% worse RMSE), or partially missing HR data (13% worse RMSE with 40% of the data missing).

A novel feature of the individualized model is that it adapts the model parameters to each subject and, as such, directly accounts for subject-specific variations in thermoregulatory responses (e.g., because of acclimation or fitness level) and responses to exogenous factors (e.g., clothing and environmental conditions). This is made possible by the two elements of the model (mathematical equations and the Kalman filter). The mathematical model equations provide the physiological relationships linking T_c_ and the noninvasively measured variables HR and T_s_. In turn, the adjustable parameters in the equations provide the degrees of freedom to customize the model to an individual based on the measured HR and T_s_. This is achieved through the Kalman filter. As it attempts to minimize the errors between the measured and the model-estimated values of HR and T_s_ after each measurement, it changes the equation parameters. In doing so, it gradually adapts the model parameters, individualizing the model to best represent the measured data and, hence, improving the estimates of T_c_.

**Fig. E1.**
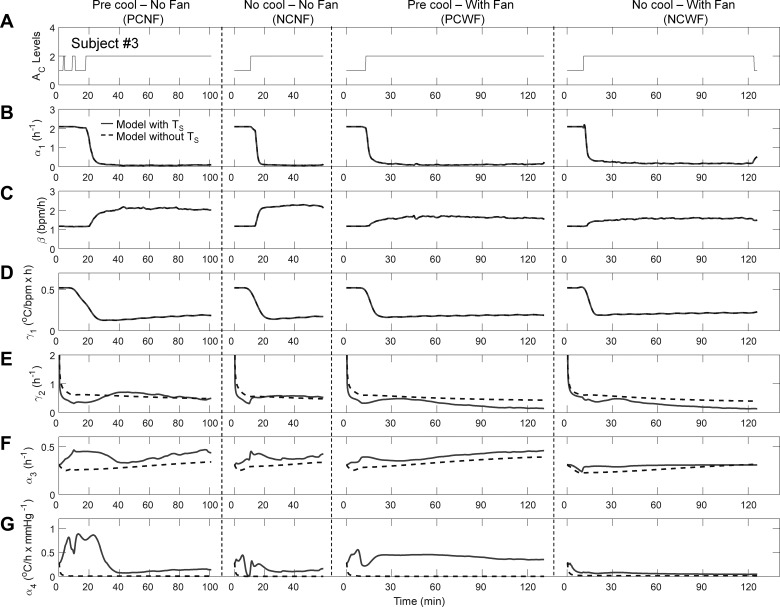
Time profiles of the six adjustable model parameters (α_1_, β, γ_1_, γ_2_, α_3_, and α_4_) in the individualized model for the same subject from *study 3* shown in Fig. 3. *A*: activity profiles of the subject while performing the cycle task under four experimental conditions at a T_a_ of 30°C and a RH of 50%. *B*–*G*: time profiles of the six parameters.

Here, we specifically demonstrated that the model can learn the variations in the thermoregulatory responses of the same subject under two different environmental conditions (22 and 40°C) by reducing α_3_ (the rate of convective heat loss from the skin to the environment) more at 40°C than at 22°C. Similarly, the model adapted to subjects performing the same activity under four different experimental conditions in which the subjects’ baseline T_c_ (via precooling) or skin heat loss (via fan airflow) was varied during the task. The model accounted for these variations by adjusting γ_2_ (the rate of heat transfer from the core to the skin compartment to partially account for precooling) and α_4_ (the rate of evaporative heat loss to partially account for fan airflow), in addition to activity-related adjustments to other parameters (α_1_, β, and γ_1_; see Figs. E1, *E* and *G*, in appendix e). This strongly suggests that adaptation of model parameters is necessary to improve T̂_c_ and thereby highlights the potential limitations of existing data-driven algorithms, which do not provide for parameter updates (5, 49), and of detailed higher-order thermoregulatory models in which a large number of parameters makes model adaptation difficult (17–19, 22).

Further analyses of the data suggest that the model can adapt to individual differences, regardless of the factors involved (e.g., sex, age). Using data from *study 1*, we performed eight pairwise comparisons across three factors: sex (*study 1* included 42 men and 18 women), environment (T_a_ = 22 or 40°C), and heat tolerance condition (6 men and 8 women were heat intolerant). Our analyses revealed that the average RMSE difference across these comparisons was <0.06°C (this value was achieved for heat-intolerant women vs. heat-intolerant men at T_a_ = 40°C). We also evaluated the effect of age on model performance by dividing the subjects from all three studies into two age groups, using a cutoff of 30 yr. The average difference in RMSE (0.02°C) was not statistically significant (Wilcoxon rank-sum test, *P* = 0.98). These analyses indicate the broad applicability of the individualized model in spite of interindividual differences.

Interestingly, the model without T_s_ provided T̂_c_ values that were indistinguishable from those provided by the model that used T_s_ measurements. This was achieved even though, in the former model, T̂_s_ values deviated considerably from their corresponding measurements (Figs. 2*C*–4*C*), suggesting that large errors in T̂_s_ did not translate into large errors in T̂_c_. This can be attributed to the fact that, in the studies investigated here, the average gradient between the measured T_c_ and T̂_s_ was similar for the two variants of the model [model with T_s_: 3.30 (SD = 2.63)°C; model without T_s_: 2.94 (SD = 1.23)°C]. As a result, the converged values of γ_2_ (the rate of heat transfer between the core and skin compartments) differed, on average across all subjects and experimental conditions, between the two model variants by only 16 (SD = 13)%. In certain scenarios, such as in cold conditions when the measured T_s_ is likely to be lower, the gradient between T_c_ and T_s_ could be greater. In this case, the model without T_s_ would then generate an erroneous estimate of γ_2_, which in turn would reduce its ability to accurately estimate T_c_. Under such scenarios, we expect that T_s_ measurements would provide additional information to correct the γ_2_ estimate and improve the accuracy of T̂_c_. We emphasize that this limitation does not apply to the full model but only to the model that does not use T_s_ measurements.

The data sets we used were limited to those from experiments in which healthy subjects performed two types of activity (walking on a treadmill and cycling) in environmental conditions varying between 20 and 40°C at 40% or 50% relative humidity. Under real-world conditions, however, men and women differing in many factors [e.g., age, body composition, fitness level, presence or absence of an illness that can induce heat injury (11, 55, 57), acclimation level, clothing, and hydration status] perform free-ranging activities of varying intensity. Studies that systematically vary several of the aforementioned factors and include subjects with T_c_ values well within the zone of clinical relevance could further serve to validate our model. Nevertheless, our basic premise is that the physiological variables (HR and T_s_) measured from individuals are reflective of subject-specific differences in the aforementioned factors. For example, an elevated HR may reflect a change in an individual’s metabolic activity in response to illness, whereas a change in T_s_ indicates some change in environmental condition. Similarly, A_c_ reflects the intensity level of physical activity. Thus, the model can estimate T_c_ in different scenarios because the inputs capture subject-specific and environmental factor differences and because the model automatically accounts for these differences by minimizing the errors between the measured and model-estimated values of HR and T_s_. Indeed, here we demonstrated that the model accurately estimates the same individual’s T_c_ under different environmental or experimental conditions and is robust to real-world operational issues. Importantly, we showed that the model performs similarly despite interindividual differences in attributes, such as age and sex.

### 

#### Conclusion.

We used a Kalman filter algorithm that relies on noninvasive measurements of physiological signals and environmental variables to adapt a mathematical model to provide real-time subject-specific individualized T_c_ estimates. The individualized model provided accurate T_c_ estimates even under real-world operational conditions, such as when measurements were unavailable, only partially available, or unreliable. Importantly, we demonstrated that the model-estimated individualized T_c_ corroborated the findings from the three studies analyzed here, suggesting that it can serve as a surrogate for measurements obtained by invasive T_c_ sensors and thereby allow for continuous monitoring of an individual’s T_c_ for an extended period. We are currently integrating the individualized model into a hardware/software system to provide early warning of an impending heat injury at an individual level. Specifically, we are combining physiological responses, measured by a fitness-tracking wristwatch and wirelessly transmitted to a smartphone that houses the individualized model, with a previously developed algorithm to predict T_c_ 20 min ahead of the current time (36). In conclusion, if the individualized model provides accurate T_c_ estimates, the early warning system’s ability to predict an impending rise in T_c_ with sufficient lead time should allow users to proactively intervene and reduce the risk of heat injuries.

## GRANTS

This study was supported by the Military Operational Medicine Research Area Directorate of the U.S. Army Medical Research and Materiel Command, Ft. Detrick, MD.

## DISCLOSURES

The opinions and assertions contained herein are the private views of the authors and are not to be construed as official or as reflecting the views of the U.S. Army or of the U.S. Department of Defense. This paper has been approved for public release with unlimited distribution. The authors have no conflicts of interest. No conflicts of interest, financial or otherwise, are declared by the authors.

## AUTHOR CONTRIBUTIONS

S.L., V.R., and J.R. conceived and designed research; S.L. and J.R. analyzed data; S.L. interpreted results of experiments; S.L. prepared figures; S.L. drafted manuscript; S.L., T.O., and J.R. edited and revised manuscript; J.B.K., R.Y., I.K., Y.E., and S.A.M. performed experiments; J.R. approved final version of manuscript.

## Figures and Tables

**Fig. A1.**
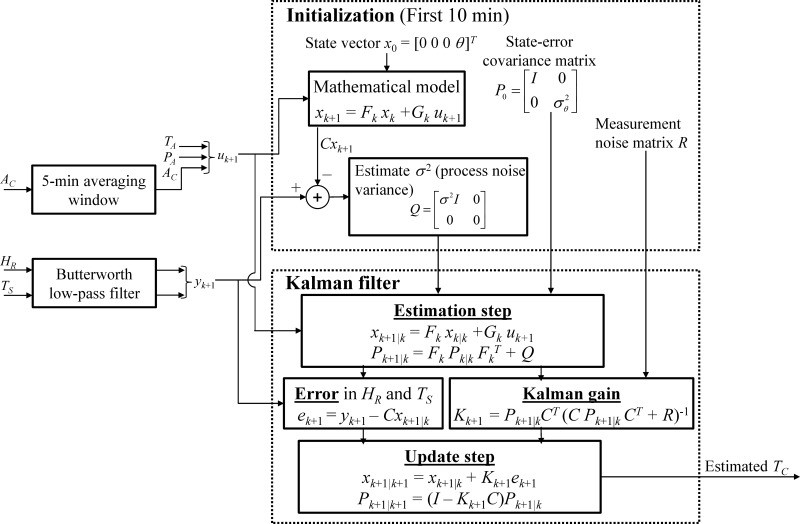
Flowchart showing the initialization and subsequent steps of the Kalman filter algorithm for real-time T_c_ estimation (see text for symbol definitions). We initialized the algorithm with θ, σθ^2^, the HR and T_s_ measurement noise variances (matrix ***R***), and the process noise variance σ^2^ estimated from the first 10 min of data for each subject. After initialization, the algorithm proceeded by using *u_k_*_+1_ to drive the mathematical model to estimate HR and T_s_ (estimation step). Next, by scaling the error *e_k_*_+1_ between the filtered measurements and the model-estimated HR and T_s_ by the Kalman gain ***K****_k_*_+1_, the algorithm updated the model parameters and the T_c_ estimates at each time index (update step) until the end of the measured time series data.

**Fig. D1.**
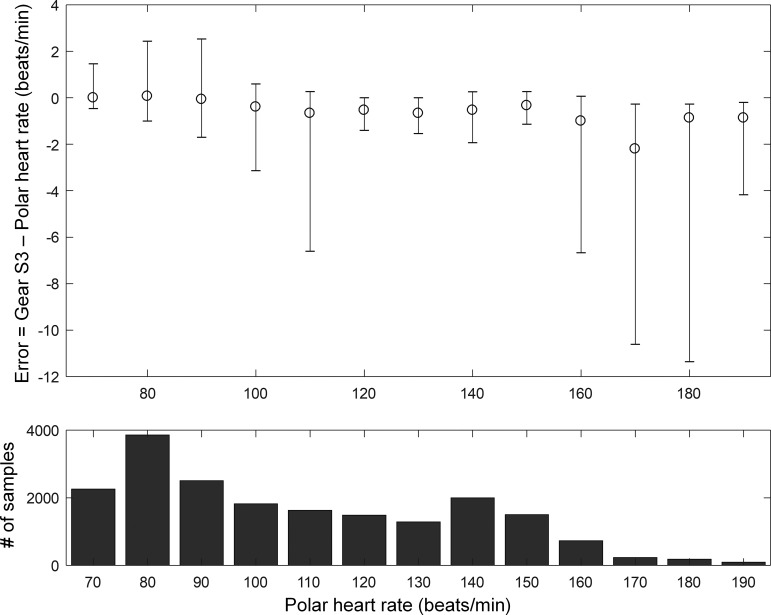
HR error characteristics of the Samsung Gear S3 smartwatch. We compared the Gear S3 HR data obtained from 83 h of exercise recordings against the gold-standard polar chest-strap data (sampled at 15-s intervals). *Top*, medians and interquartile ranges of the errors plotted for each of the 10 beats/min bins. *Bottom*, no. of HR samples in each bin.

**Fig. F1.**
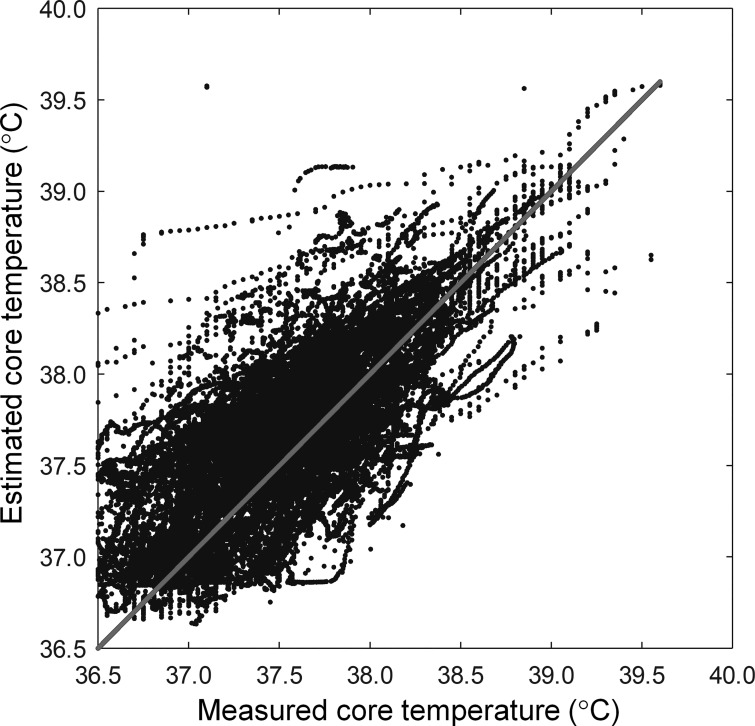
Scatterplot comparing the estimated T_c_ against the corresponding measurements across all 166 subjects and experimental conditions (42,026 data points from 247 time profiles). The solid line is the line of equality. The Pearson’s correlation coefficient was 0.72.

## APPENDIX A: THE KALMAN FILTER ALGORITHM

To implement the Kalman filter algorithm, we needed to convert the continuous-time nonlinear model (*Eqs. 1**–**3* in *Mathematical model* in methods) into a discrete linear model. To discretize the model, we noted that *Eqs. 1**–**3* are of the form ***ẋ*** *= f*(***x***,***u***), with states *x* = [ΔHR ΔT_c_ ΔT_s_ θ]^T^, where θ = **ϕ**^1/2^, with **ϕ** representing the vector of the six adjustable model parameters, *f*(⋅) denoting a set of nonlinear functions, and $u={\left[{\text{A}}_{\text{c}}^{4}{\text{T}}_{\text{a}}{\text{P}}_{\text{a}}\right]}^{\text{T}}$. We set θ = **ϕ**^1/2^ to ensure nonnegative parameter values. We then computed the Jacobian of *f*(***x***,***u***) (47) and discretized the results to obtain a linear time-varying model (10). The discrete model had state equations

***x****_k+_*_1_ *=* ***F****_k_* ***x****_k_ +* ***G****_k_* ***u****_k_*_+1_ and output equations *y_k_ =* ***C x****_k_* + ***y***_0_, where ***F****_k_* and ***G****_k_* denote the discrete linearized state-transition and input matrices, respectively, obtained at each time index *k*, ***C*** denotes the matrix that outputs the estimated ΔHR and ΔT_s_ signals, and ***y***_0_ = [HR_0_ T_s0_]^T^ represents the vector of the mean values of HR and T_s_ from the initial 10 min of data that were used to initialize the model.

The flowchart in Fig. A1 depicts the initialization of, and sequence of steps involved in, the Kalman filter algorithm. As described in methods, we initialized the algorithm with parameter values θ (which is part of the initial state vecto*r* ***x***_0_) obtained from previously published studies and their corresponding variances σθ^2^ (which is part of the initial state-error covariance matrix ***P***_0_). In addition, we used HR and T_s_ measurements obtained from the first 10 min of data to estimate the noise variances (which comprise matrix ***R***) and the systemic uncertainty between estimated and measured values of HR and T_s_ (σ^2^ in matrix ***Q***). Using each subject’s first 10 min of data, we estimated σ^2^. To do so, we first filtered the measured HR and T_s_ in real time, using a second-order causal low-pass Butterworth filter with a cut-off frequency of 3.3 mHz, to reject noise while preserving the frequency band that overlaps with the measured T_c_. We then used a 5-min moving average of the measured A_c_, the measured T_a_, and P_a_ (computed from the measured T_a_ and RH) to drive the mathematical model and computed the error between the filtered HR and T_s_ data and the corresponding model estimates. This error, which is linearly related to the systemic uncertainty in the model states, allowed us to estimate σ^2^ by solving the resulting linear least-squares problem (7).

After initialization, the Kalman filter algorithm proceeded in the following manner. At each 15-s discrete time interval, the algorithm used a 5-min moving average of the measured (or computed) A_c_, the measured T_a_, the P_a_ at the present time index *k* + 1, and the model parameters obtained up to time index *k* to estimate the model states ***x****_k_*_+1|_*_k_* (Fig. A1, estimation step). In the estimation step, the algorithm also estimated the state-error covariance matrix ***P****_k_*_+1|_*_k_*, using the model parameters up to time index *k* and the process noise matrix ***Q***. Subsequently, the algorithm computed the Kalman gain ***K****_k_*_+1_. Next, the algorithm used the error *e_k_*_+1_ between the filtered measurements (***y****_k_*_+1_) and the estimated HR and T_s_ (***Cx****_k_*_+1|_*_k_*) to update the T_c_ estimate (an element of the state vector ***x****_k_*_+1|_*_k_*_+1_), the model parameters, and the state-error covariance matrix ***P****_k_*_+1_*_|k_*_+1_ (Fig. A1, update step). In situations where HR or T_s_ measurements were temporarily unavailable (i.e., missing measurements), we assumed that the noise characteristics of the error at time point *k* + 1 would not be drastically different from the previous time point *k* and, hence, set *e_k+_*_1_ to *e_k_* in the update step. The algorithm repeated this procedure for each time step until the end of the time series data.

## APPENDIX B: PEAK T_c_ STATISTICS

To assess the individualized model’s ability to estimate the timing and magnitude of the measured peak T_c_ in each profile, we computed the following three statistics: *1*) the absolute time difference between the measured and model-estimated peak T_c_, *2*) the absolute difference between the magnitude of the measured peak T_c_ and the model estimate at the same time point, and *3*) the absolute difference between the magnitudes of the measured and model-estimated values of peak T_c_. Table B1 reports the values of these statistics for all subjects in each of the three studies.

## APPENDIX C: MODIFIED KALMAN FILTER ALGORITHM TO HANDLE UNAVAILABLE T_s_

The Kalman filter algorithm described above cannot be directly applied to the case where T_s_ is not measured. This is because the model parameters, which depend on T_s_ measurements (γ_1_, γ_2_, α_3_, and α_4_, see *Eqs. 2* and *3* in *Mathematical model* in methods), cannot be adapted to the individual without such measurements. Hence, for running the model without T_s_ measurements, we modified the update step of the Kalman algorithm by setting *e_k_*_+1_ = T̂_s_*_k_*_+1_ – T̂_s_*_k_*, i.e., we used the difference in T̂_s_ between the present time point *k* + 1 and the previous time point *k* as a surrogate for *e_k_*_+1_. This enabled the model parameters to be adapted to the individual despite the absence of T_s_ measurements.

## APPENDIX D: SIMULATING NOISE-LADEN HR MEASUREMENTS

To simulate noise added to the HR data, we first obtained the error characteristics of the Gear S3 smartwatch (Samsung Electronics America) by comparing the HR measurements of the watch against those of a gold-standard chest-strap device (Polar H7; Polar Electro Oy) under different physical activities for a total of 83 h of recordings sampled at 15-s intervals. We binned the HR measurements from the chest-strap device into 10-beat intervals ranging from 70 to 190 beats/min, with each bin comprising HR values within 5 beats/min of the bin center (e.g., the bin centered at 70 beats/min included data ranging from 65 to 75 beats/min). We then computed the error distribution for each HR bin. Thus, we obtained 13 error distributions, one for each of the 13 HR bins (70, 80, . . . , 190, all in units of beats/min). Figure D1 shows the error characteristics of the Gear S3 smartwatch.

To add noise to the HR data, we started with the time profile of the measured HR for a given subject. Next, for each HR value from the time profile, we identified the corresponding HR bin, randomly sampled an error value from the cumulative distribution function of the particular error distribution corresponding to that bin, and added that value to the HR data. For example, if the true HR value was 83 beats/min, we selected the error distribution for the HR bin centered at 80 beats/min, randomly sampled an error value from the cumulative distribution function of that error distribution (e.g., 3 beats/min), and added that value to the true HR value to obtain the noise-added value of 86 beats/min. We repeated this procedure for all HR values throughout the time profile to obtain one noise-added HR realization. For each time profile for a given subject, we performed 100 such realizations.

## APPENDIX E: TIME PROFILES OF THE SIX ADJUSTABLE MODEL PARAMETERS FOR A SUBJECT FROM *STUDY 3*

Figure E1 shows the time profiles of the six model parameters for the subject shown in Fig. 3 (*study 3*, *subject 3*). Apart from the activity-related changes that affect all parameters, changes in the experimental condition affect parameters γ_2_ and α_4_. Specifically, parameter γ_2_, the rate of heat transfer from the core to the skin compartment, responds to the difference between T_c_ and T_s_ (see *Mathematical model*, *Eqs. 2* and *3* in methods) and, hence, is affected slightly differently between precooling (T_c_ – T_s_ ~6.5°C initially) and no-cooling (T_c_ – T_s_ ~7.0°C initially; see Fig. 3, *C* and *D*, in results) conditions. Figure E1*E* shows that γ_2_ is slightly lower in the PCNF condition (~30 min from the beginning when the first T_c_ measurement is shown in Fig. 4) than in the NCNF condition (~15 min from the beginning when the first T_c_ measurement is shown in Fig. 4). Note, however, that in the other two conditions (PCWF and NCWF) there is no difference in the value of γ_2_. Thus, although precooling affects γ_2_, the effect is masked by variation in T_s_ between experimental conditions.

The rate of sweat evaporation, α_4_, depends on the difference between the vapor pressures of water in the skin and the environment (P_s_ – P_a_, see *Eq. 3* in *Mathematical model* in methods). Although both factors are indirectly affected by airflow, the difference P_s_ – P_a_ was very low in these experimental conditions (because they depend on T_s_ and T_a_, both of which were around 30°C). Hence, we expected only a slightly larger value of α_4_ in the fan airflow conditions (PCWF and NCWF) compared with the other two conditions, but the value was higher only in the PCWF condition (Fig. E1*G*).

## APPENDIX F: SCATTERPLOT OF ESTIMATED vs. MEASURED T_c_ VALUES ACROSS ALL 166 SUBJECTS AND EXPERIMENTAL CONDITIONS

Figure F1 shows a scatterplot comparing the estimated T_c_ against the corresponding measurements across all 166 subjects and experimental conditions (42,026 data points from 247 time profiles). The Pearson’s correlation coefficient between the measured and estimated T_c_ was 0.72, indicating good agreement across the entire temperature range. Note that temporal information critical for continuously monitoring T_c_ during physical activity is lost in such a plot. Furthermore, the plot masks information about the ability of the model to be individualized to a subject and is only presented here to indicate overall agreement between T_c_ estimates and T_c_ measurements.

### Symbols

#### Abbreviations

α_1_: Rate constant for the heart rate signal (h^−1^)
α_2_: Thermoregulatory rate constant for the core body temperature signal (h^−1^)
α_3_: Rate of convective heat transfer from the skin compartment to the environment (h^−1^)
α_4_: Rate of heat loss to the environment due to sweat evaporation (h^−1^ × °C/mmHg)
A_c_: Activity level ranging from 0 (low) to 4 (high)
β: Gain in heart rate due to physical activity (beats·min^−1^·h^−1^)
***C***: Output matrix of the state-space representation of the individualized model, which outputs the states ΔHR and ΔT_s_ from the state vector ***x***
Δ: Change from the baseline condition
*f*(***x***,***u***): A set of nonlinear functions representing the derivative of the state vector ***x*** with respect to time
***F***: Local linearization of *f*(***x,u***) with respect to ***x***
γ_1_: Rate of heat gain due to metabolic activity [°C/h × (beats/min)^−1^]
γ_2_: Rate of heat loss/gain from the core to the skin compartment (h^−1^)
***G***: Local linearization of *f*(***x*,*u***) with respect to ***u*** HR Heart rate (beats/min)
$\hat{HR}$: Model-estimated heart rate (beats/min)
*k*: Time index
***K***: Kalman gain matrix
**ϕ**: Model parameter vector [α_1_ β γ_1_ γ_2_ α_3_ α_4_]^T^
***P***: State-error covariance matrix
P_a_: Vapor pressure of water due to the heat index perceived by humans (mmHg)
P_s_: Vapor pressure of water for skin temperature (mmHg)
***Q***: Process noise variance matrix
***R***: Measurement noise matrix
RH: Relative humidity (%)
σ: SD of the process noise
σθ: SD of θ
θ: Square root of the model parameters **ϕ**^1/2^
*S*(ΔHR): Sigmoid function applied to ΔHR
T_a_: Ambient temperature (°C)
T_c_: Core body temperature (°C)
T̂_c_: Model-estimated core body temperature (°C)
T_s_: Skin temperature (°C)
T̂_s_: Model-estimated skin temperature (°C)
***u***: Input vector ${\left[{\text{A}}_{\text{c}}^{4}{\text{T}}_{\text{a}}{\text{P}}_{\text{a}}\right]}^{\text{T}}$
***x***: State vector [ΔHR ΔT_c_ ΔT_s_ θ]^T^
***y***: Output vector [HR T_s_]^T^

### Abbreviations

#### Abbreviations

HTT: Heat tolerance test
MET: Metabolic equivalent ratio of oxygen consumed during a specific physical activity to that at rest
NCNF: No precooling and no fan airflow during cycle ergometer task
NCWF: No precooling but with fan airflow during cycle ergometer task
PCNF: Precooling and no fan airflow during cycle ergometer task
PCWF: Precooling but with fan airflow during cycle ergometer task
RMSE: Root mean squared error
SD: Standard deviation

## Appendix

**Table 4.** Comparison of the measured T_c_ and the corresponding model estimate relating to the major findings in study 2

Heat-tolerant subjects (Ta = 40°C)		Heat-intolerant subjects (T_a_ = 40°C)	
Data	Model	Data	Model
37.90 (0.26)	38.06 (0.26)	38.53 (0.27)*	38.58 (0.23)*
0.22 (0.12)	0.23 (0.15)	0.59 (0.19)*	0.51 (0.21)*

Entries indicate mean values with SD in parentheses; *n* = 73 heat-tolerant men and 23 heat-intolerant men.

* *P* < 0.05, Wilcoxon rank sum test comparing heat-tolerant vs. heat-intolerant subjects.

**Table B1.** Performance of the individualized model in estimating the T_c_ of subjects from the three studies under different experimental conditions

Study (No. of Subjects)	Condition	No. of Profiles/Condition	Time Difference between max T_c_ and max T̂_c_, min	|max T_c_ – T̂_c_|, °C	|max T_c_ – max T̂_c_|, °C
1 (60)*	22°C	60	35 (31)	0.26 (0.18)	0.26 (0.19)
	40°C	60	20 (21)	0.26 (0.20)	0.30 (0.26)
2 (96)*	40°C	96	17 (23)	0.24 (0.18)	0.25 (0.19)
3 (10)^†^	NCNF	7	2 (2)	0.29 (0.15)	0.26 (0.16)
	PCNF	10	3 (2)	0.64 (0.37)	0.57 (0.38)
	NCWF	7	3 (4)	0.17 (0.13)	0.17 (0.10)
	PCWF	7	4 (5)	0.37 (0.31)	0.39 (0.30)
Total (166)		247	20 (24)	0.27 (0.20)	0.28 (0.21)

Reported values are means with SD in parentheses. The absolute time difference between the measured T_c_ peak (max T_c_) and the model-estimated T_c_ peak (max T̂_c_), the absolute error between max T_c_ and T̂_c_ at the same time point, and the absolute error between max T_c_ and max T̂_c_ are shown.

* *Study 1* was conducted under two different T_a_ conditions, whereas *study 2* was conducted at 40°C. In both studies, the relative humidity was set to 40%.

† *Study 3* was conducted at a T_a_ of 30°C at a relative humidity of 50%.
